# Construction and verification of atopic dermatitis diagnostic model based on pyroptosis related biological markers using machine learning methods

**DOI:** 10.1186/s12920-023-01552-5

**Published:** 2023-06-17

**Authors:** Wenfeng Wu, Gaofei Chen, Zexin Zhang, Meixing He, Hongyi Li, Fenggen Yan

**Affiliations:** 1 The Second Clinical College, Guangzhou University of Chinese Medicine, Guangzhou, China; 2 Zhongshan Hospital of Traditional Chinese Medicine Affiliated to Guangzhou University of Traditional Chinese Medicine, Zhongshan, China; 3 The First Clinical College, Guangzhou University of Chinese Medicine, Guangzhou, China; 4 Department of Dermatology, The Second Affiliated Hospital of Guangzhou University of Chinese Medicine (Guangdong Provincial Hospital of Chinese Medicine), Guangzhou, China; 5 Guangdong Provincial Key Laboratory of Chinese Medicine for Prevention and Treatment of Refractory Chronic Diseases, The Second Affiliated Hospital of Guangzhou University of Chinese Medicine (Guangdong Provincial Hospital of Chinese Medicine), Guangzhou, China; 6 Guangdong-Hong Kong-Macau Joint Lab on Chinese Medicine and Immune Disease Research, Guangzhou, China; 7 State Key Laboratory of Dampness Syndrome of Chinese Medicine, The Second Affiliated Hospital of Guangzhou University of Chinese Medicine, Guangzhou, China

**Keywords:** Machine learning, Pyroptosis, Atopic dermatitis, Disease diagnosis, Immune cells infiltration

## Abstract

**Objective:**

The aim of this study was to construct a model used for the accurate diagnosis of Atopic dermatitis (AD) using pyroptosis related biological markers (PRBMs) through the methods of machine learning.

**Method:**

The pyroptosis related genes (PRGs) were acquired from molecular signatures database (MSigDB). The chip data of GSE120721, GSE6012, GSE32924, and GSE153007 were downloaded from gene expression omnibus (GEO) database. The data of GSE120721 and GSE6012 were combined as the training group, while the others were served as the testing groups. Subsequently, the expression of PRGs was extracted from the training group and differentially expressed analysis was conducted. CIBERSORT algorithm calculated the immune cells infiltration and differentially expressed analysis was conducted. Consistent cluster analysis divided AD patients into different modules according to the expression levels of PRGs. Then, weighted correlation network analysis (WGCNA) screened the key module. For the key module, we used Random forest (RF), support vector machines (SVM), Extreme Gradient Boosting (XGB), and generalized linear model (GLM) to construct diagnostic models. For the five PRBMs with the highest model importance, we built a nomogram. Finally, the results of the model were validated using GSE32924, and GSE153007 datasets.

**Results:**

Nine PRGs were significant differences in normal humans and AD patients. Immune cells infiltration showed that the activated CD4+ memory T cells and Dendritic cells (DCs) were significantly higher in AD patients than normal humans, while the activated natural killer (NK) cells and the resting mast cells were significantly lower in AD patients than normal humans. Consistent cluster analysis divided the expressing matrix into 2 modules. Subsequently, WGCNA analysis showed that the turquoise module had a significant difference and high correlation coefficient. Then, the machine model was constructed and the results showed that the XGB model was the optimal model. The nomogram was constructed by using HDAC1, GPALPP1, LGALS3, SLC29A1, and RWDD3 five PRBMs. Finally, the datasets GSE32924 and GSE153007 verified the reliability of this result.

**Conclusions:**

The XGB model based on five PRBMs can be used for the accurate diagnosis of AD patients.

**Supplementary Information:**

The online version contains supplementary material available at 10.1186/s12920-023-01552-5.

## Background

Atopic dermatitis (AD) is a common, intractable pruritic and chronic inflammatory skin disease that is often associated with other atopic diseases such as asthma and allergic rhinitis [1, 2]. According to the Global Burden of Disease Study, the age-standardized incidence rate of AD was 327.91 for every 100,000 persons one year and had the highest disability-adjusted life year burden among all skin diseases, ranking in the top 15 among all non-fatal diseases [3, 4]. Repeated itch-scratch cycles and sleep disturbances reduce patients’ quality of life and increase the risk of metabolic, psychiatric, and cardiovascular diseases [5]. Unfortunately, the pathogenesis of AD is complex and multi-factorial, including genetic susceptibility, skin barrier dysfunction, and altered immune response, which pose significant challenges to the medical community [6].

Increasing shreds of evidence showed that pyroptosis has great research prospects in inflammatory skin diseases [7]. Pyroptosis is a newly discovered form of inflammatory-associated programmed cell death, including canonical and non-canonical pathways [8] that regulate cell fate and determine disease progression [9]. Of note, the canonical pathway is generally initiated by pathogen-associated and damaged-associated molecular patterns, which subsequently induces apoptosis-associated speck-like protein containing a caspase-recruiting domain (ASC) recruitment to form inflammasomes. Then, procaspase-1 is cleaved by inflammasomes to activate caspase-1, and activated caspase-1 results in the cleavage of gasdermin D (GSDMD) and the maturation of IL-1B and IL-18 [9]. While in the noncanonical pathway, lipopolysaccharide can activate caspase-4/5/11 to induce pyroptosis by cleavage of GSDMD [10]. The role of PRGs in AD has been elucidated in recent years, especially the inflammasome [7]. Studies showed that the expression of inflammasome NLR Family Pyrin Domain Containing 3 (NLRP3) and absent in melanoma-2 (AIM2) was increased in AD and was related to epidermal inflammation [11, 12]. Moreover, NLRP3, caspase-1, and IL-1B, involved in the pyroptosis canonical pathway, might be related to mental disorders of AD [13]. These findings indicated that PRGs may act as a pivotal part of AD and identifying more comprehensive potential PRBMs can provide a novel perspective on the diagnosis and treatment of AD.

Additionally, immune dysregulation is one of the major pathogenic mechanisms of AD. Immune cells such as dendritic cells (DC) and CD4+ memory T cells could secrete related inflammatory factors, which played a significant role in the occurrence and progression of AD [14]. The expression of TH2, TH22, TH1, and TH17 cell-related mediators in the skin of patients increased with the severity of the disease [15]. Through modulating allergen-induced IgE-mediated mast cell ROS generation and oxidation of calmodulin kinase II, the DC immunoreceptor could alleviate the inflammation of AD [16]. Be noted, the correlation between PRGs and immune cells infiltration has been studied in some allergic diseases, such as urticaria and asthma [17, 18]. Nonetheless, their mechanisms in AD are still unknown.

Machine learning (ML) is a technique that allows computers to perform complex tasks [19]. Commonly used ML models include SVM, RF, XGB and GLMs, and the combination of multiple models can improve the accuracy of diagnosis [20, 21]. Despite the widespread use of ML models, the application of ML models in the diagnosis of AD based on PRBMs has yet to be reported. Therefore, in this study, the PRBMs related to AD were identified and used to construct diagnostic models used for the accurate diagnosis of AD.

## Material and methods

### Data downloading and processing

**Fig. 1:**
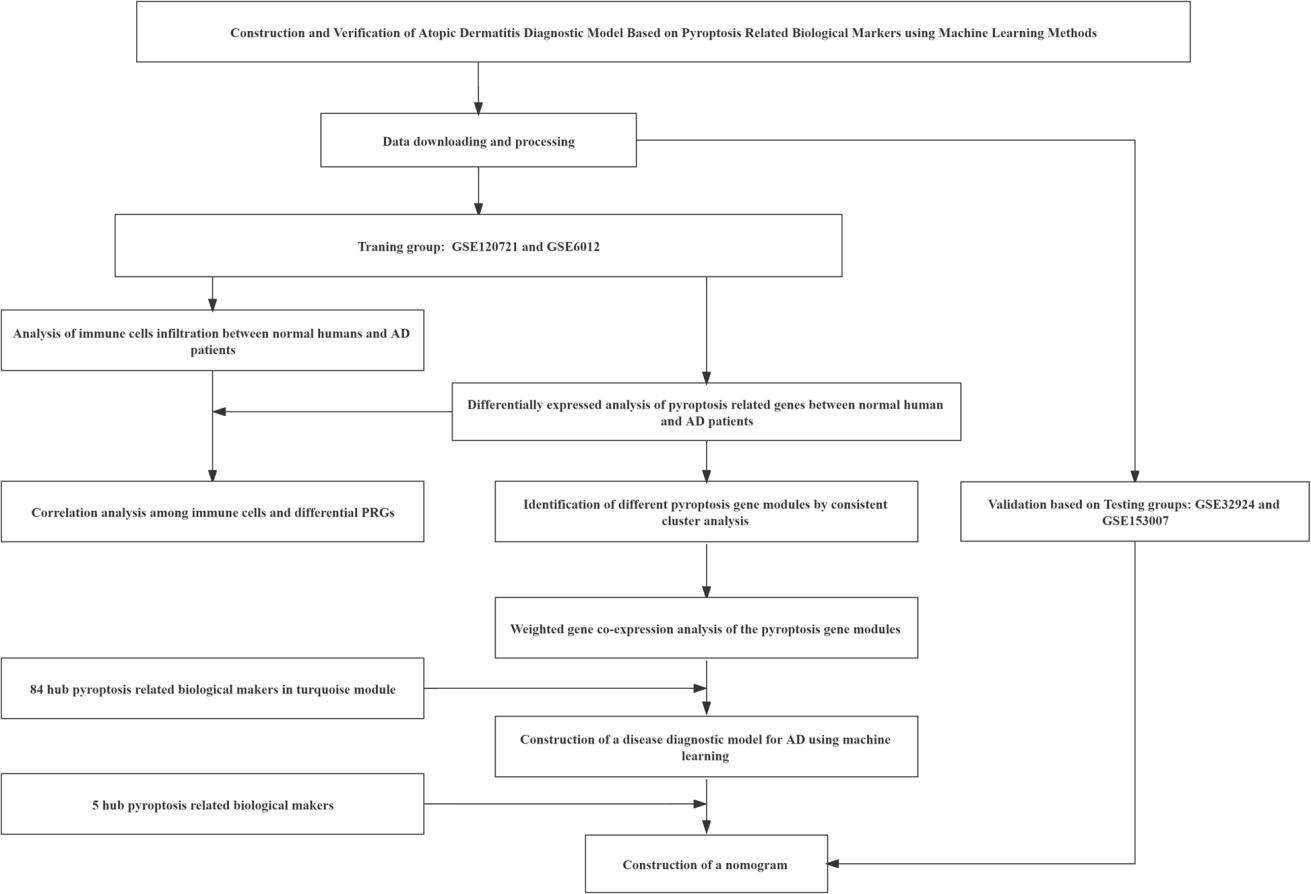
Flow of this study

The flow of this study was showed in Fig. 1. The chip data of GSE120721, GSE6012, GSE32924, and GSE153007, and its corresponding platform files GPL570, GPL96, GPL570, and GPL6480 were acquired from the GEO database (https://www.ncbi.nlm.nih.gov/geo/). Then, The probe matrix of GSE120721, GSE6012, GSE32924, and GSE153007 was transformed into gene matrix by using Perl language. The data of GSE120721 and GSE6012 were combined and standardized processed served as the training group by the limma package and sva package of R language software 4.2.0, while the data of GSE32924 and GSE153007 served as the testing groups. The normal humans were served as the control group, while the AD patients served as the Treat group.

### Differentially expressed analysis of pyroptosis related genes between normal human and AD patients

To acquire pyroptosis-related genes for Homo sapiens, we set the keyword as "pyroptosis" and searched the MSigDB database (http://www.gsea-msigdb.org/gsea/msigdb/). Next, we extracted the expression of the pyroptosis-related genes from the training group and used the limma package in R language software 4.2.0 to screen for differentially expressed genes (DEGs) between normal human and AD patients. Finally, we generated a heatmap and boxplot using the pheatmap package, reshape2 package, and ggpubr package in R language software 4.2.0.

### Analysis of immune cells infiltration between normal humans and AD patients

In order to explore immune cells infiltration between normal humans and AD patients, the analysis of immune cells infiltration was performed using the CIBERSORT package of R language software 4.2.0. Then, the bar graph and boxplot were plotted using the reshape2 package and ggpubr package. Furthermore, a correlation analysis between significantly differential PRGs and immune cells was performed to explore their relationship using the corrplot package of R language 4.2.0.

### Identification of different pyroptosis gene modules by consistent cluster analysis

The gene modules with different expressing levels of pyroptosis were screened by consistent cluster analysis by ConsensusClusterPlus package of R language software 4.2.0. Consistent cluster analysis was an unsupervised clustering method for classifying disease subtypes, which was able to dig out new disease subtypes or make a comparative analysis of different subtypes [22]. The different modules were divided by choosing an optimal K value, and the clearer the boundary between the modules was, the more stable the functions within the modules became. While the optimal K value selection can be achieved by the inflection point method. In addition, the expressing levels of PRGs with significant differences between modules were screened by the limma package of R language software 4.2.0. Meanwhile, the Principal Component Analysis (PCA) was used to detect the samples between the modules whether could be distinguished by the expressing levels of the PRGs.

### Weighted gene co-expression analysis of the pyroptosis gene modules

To further screen the gene modules with the most significant difference and the highest correlation coefficient between the different pyroptosis gene modules, the weighted gene co-expression analysis (WGCNA) was performed using the WGCNA package of the R language software 4.2.0. On the one hand, WGCNA was a systems biological approach used to characterize the patterns of gene associations between different samples. On the other hand, it can be used to identify highly synergistic changing gene sets and alternate biomarker genes or therapeutic targets based on the internal connectivity of gene sets and the association between gene sets and phenotypes [23]. In this study, we identified different gene modules by WGCNA and screened the hub genes by setting geneSigFilter = 0.85 and moduleSigFilter = 0.95.

### Construction of a disease diagnostic model for AD using machine learning

To construct the AD disease diagnosis model, the RF, SVM, XGB, and GLM machine learning models were constructed using repeatedcv, svmRadial, xgbDART, and glm methods and using caret, DALEX, randomForest, kernlab, and glm packages. The ROC curve was plotted using the pROC package to assess the model reliability, and the importance of the five genes in the optimal model was calculated for the next analysis. We also performed the RF, SVM, XGB and GLM on the testing datasets GSE32924 and GSE153007.

### Construction of a nomogram

In order to construct a nomogram that was used for the diagnosis of AD patients, the rms package and rmda package of R language software 4.2.0 were employed. In this nomogram, points were calculated based on the expression of five PRBMs, and a total point was calculated based on the sum of the five points. Then, according to the risk of disease, the probability of occurrence of AD can be came out. Furthermore, the DCA decision curve was used to verify the diagnostic efficiency of the model, and the simulated curve was used to test the degree of fit of the model.

### Validation based on external datasets

The probe matrix of GSE32924 and GSE153007 were transformed to gene matrix by using platform files and Perl language. Next, pROC package was used to verify the efficiency of the model. The reliability of this model will be identified if the AUC higher than 0.6.

## Results

### Data downloading and processing

The chip data of GSE120721, GSE6012, GSE32924, GSE153007 and its’ corresponding platform files GPL570, GPL96, GPL570 and GPL6480 were acquired from GEO database. GSE120721 included 22 normal human samples and 15 AD patients samples. GSE6012 included 10 normal human samples and 10 AD patients samples. GSE32924 included 8 normal human samples and 13 AD patients samples. GSE153007 included 5 normal human samples and 24 AD patients samples. The data of GSE120721 and GSE6012 were combined and standardized as the training group by the limma package and sva package in R language software 4.2.0, which concluded 32 normal human samples and 25 AD patients samples.

### Differentially expressed analysis of pyroptosis genes between normal humans and AD patients

**Fig. 2:**
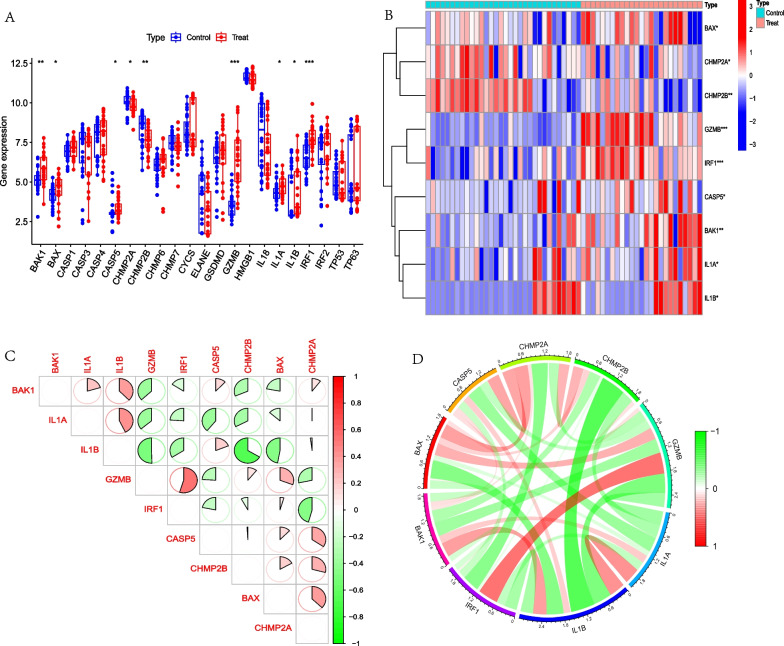
Differentially expressed analysis and correlation analysis of PRGs between normal human and AD patients. **A**. Boxplot showed that 9 PRGs were significantly different between two groups. **B**. Heatmap showed that 9 PRGs were significantly different between two groups. **C**. Heatmap showed that IL1B was negative correlation with GZMB and CHMP2B, while GZMB was positive correlation with CHMP2B. **D**. Circle diagram showed that IL1B was negative correlation with GZMB and CHMP2B, while GZMB was positive correlationwith CHMP2B. *p < 0.05; **p < 0.01; ***p < 0.001

By setting the key word as “pyroptosis” and source organism as “homo sapiens”, 27 PRGs were acquired from the MSigDB Database (Additional file 1: Table S1). Then, the expression of 22 PRGs was extracted from the training group and differentially expressed analysis was carried out between normal humans and AD patients. The result showed that 9 of 22 PRGs were significantly difference in two groups, among the expression of BAK1, BAX, CASP5, GZMB, IL1A, IL1B, IRF1 were higher in AD patients than in normal humans, while CHMP2A, CHMP2B were lower in AD patients than in normal humans. Figure 2A–B. Correlation analysis showed that IL1B was negative correlation with GZMB and CHMP2B, while GZMB was positive correlation with CHMP2B. Figure 2C–D.

### Analysis of immune cells infiltration between normal humans and AD patients

**Fig. 3:**
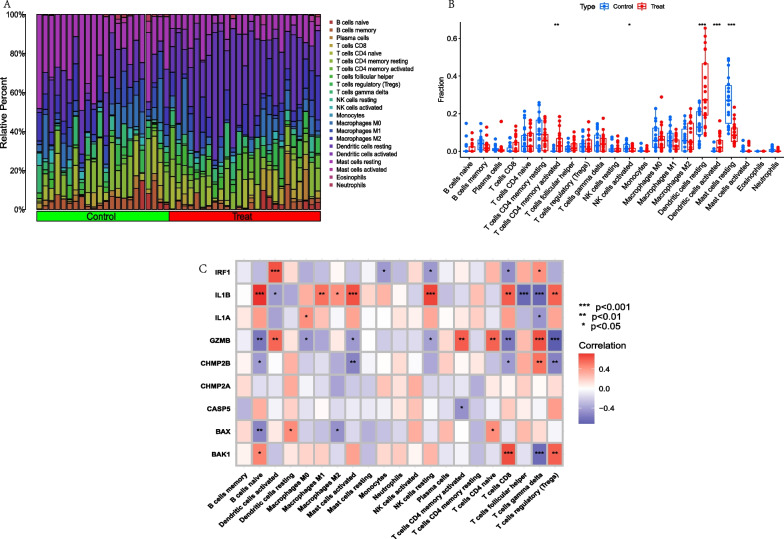
Immune cells infiltration analysis between normal human and AD patients and correlation analysis among 9 PRGs and immune cells. **A**. Bar graph of immune cells infiltration between normal human and AD patients. **B**. Boxplot showed that 5 immune cells were significantly different between two groups. **C**. Heatmap of correlation showed that GZMB was positively correlated with the DCs activated and T cells CD4 memory activated and IL1B was negatively related to DCs activated. *p < 0.05; **p < 0.01; ***p < 0.001

In order to explore immune cells infiltration between normal patients and AD patients, immune cells infiltration analysis was performed using the CIBERSORT package of R language software 4.2.0. Furthermore, the relationship analysis between 9 PRGs and immune cells was also conducted. The results showed that the activated CD4+ T cells, the resting and activated dendritic cells were significantly higher in AD patients than in normal human, while the activated NK cells and resting mast cells were significantly lower in AD patients than in normal humans. Figure 3A–B. Correlation analysis showed that IL1B was associated to 10 immune cells, which was significantly positive correlation with B cells naive, Macrophage M1, Macrophage M2, Mast cells activated, NK cells resting, T cells CD8 and T cells regulatory (Tregs) and negative correlation with the activated dendritic cells, T cells follicular helper and T cells gamma delta. While GZMB was significantly positive correlation with Dendritic cells activated, T cells CD4 memory activated, T cells CD4 naive and T cells gamma delta, and was negative correlation with the B cells naive, Macrophages M0, Mast cells activated, NK cells resting, T cells CD8 and T cells regulatory (Tregs). CHMP2B was significantly positive correlation with T cells gamma delta, and was negative correlation with the B cells naive, Mast cells activated, T cells CD8 and T cells regulatory (Tregs). Figure 3C.

### Identification of different pyroptosis gene modules by consistent cluster analysis

**Fig. 4:**
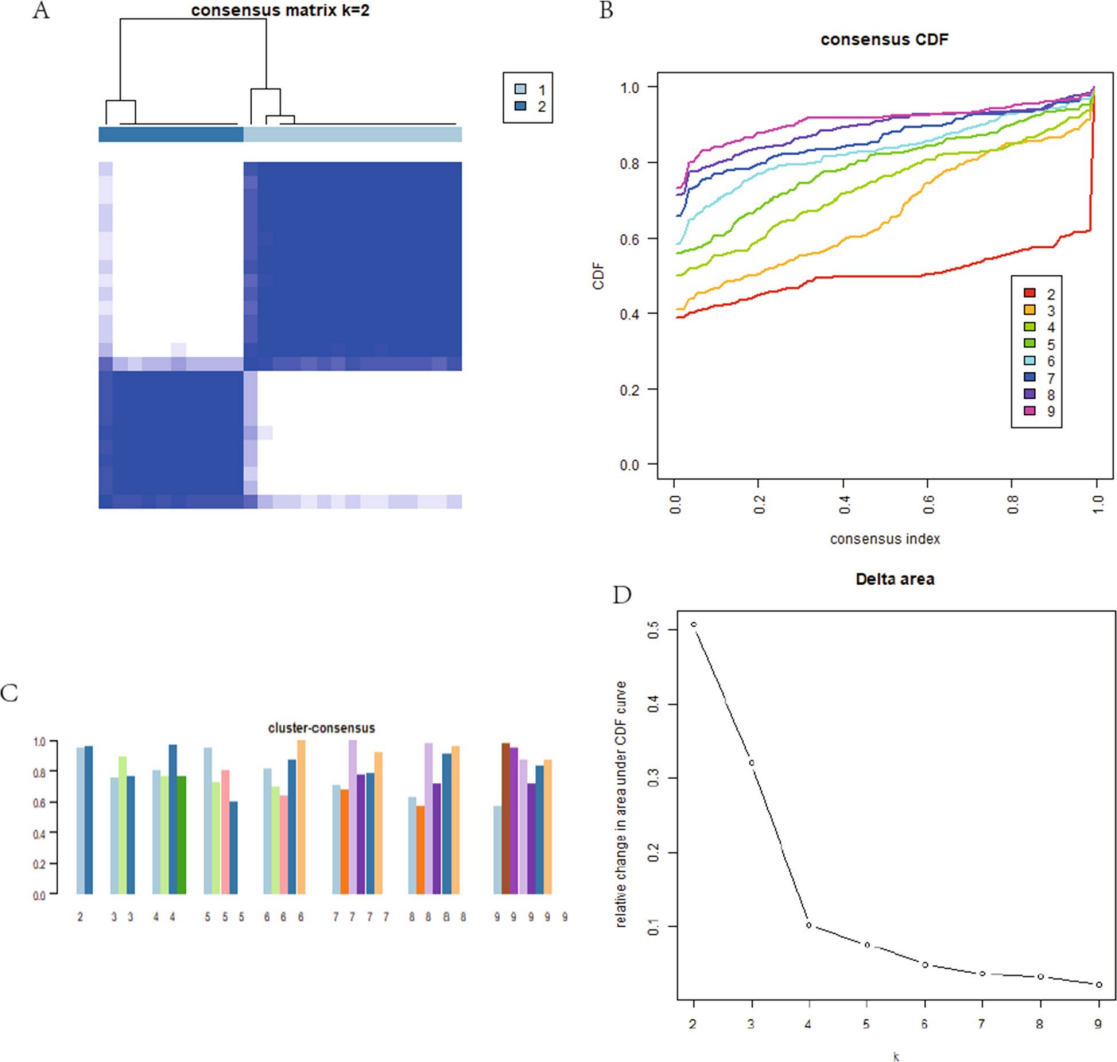
Identification of different pyroptosis gene modules by consistent cluster analysis. **A**. 2 modules were divided according to the optimal K value. **B**. Consensus index and CDF curve. **C**. Cluster consensus showed that the internal consistency greater than 0.8 when the number of module was 2. **D**. Relative change in area under CDF curve

**Fig. 5:**
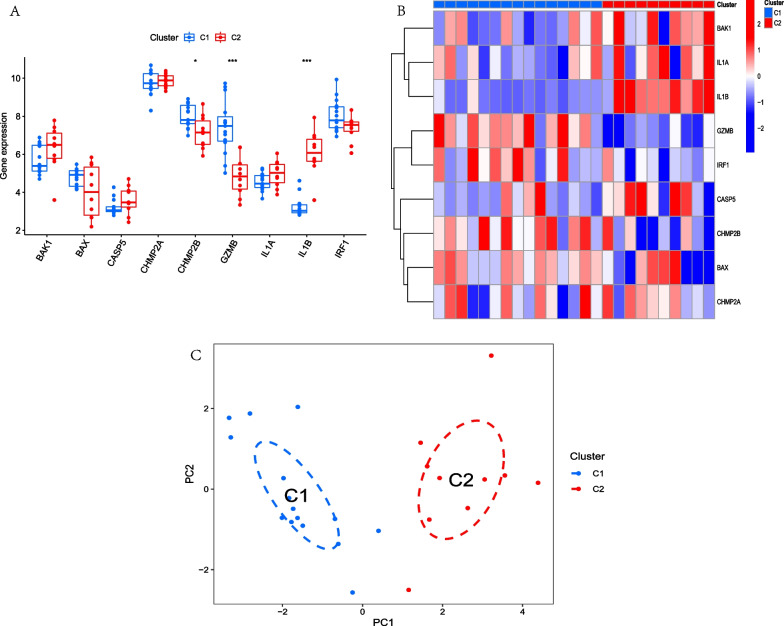
Differentially expressed analysis and PCA analysis of PRGs between C1 and C2 module. **A**. Boxplot showed that 3 PRGs were significantly different between two groups. **B**. Heatmap showed that 3 PRGs were significantly different between two groups. **C**. PCA analysis showed that patients were able to well distinguish between C1 and C2 modules based on the expression levels of PRGs. *p < 0.05; **p < 0.01; ***p < 0.001

**Fig. 6:**
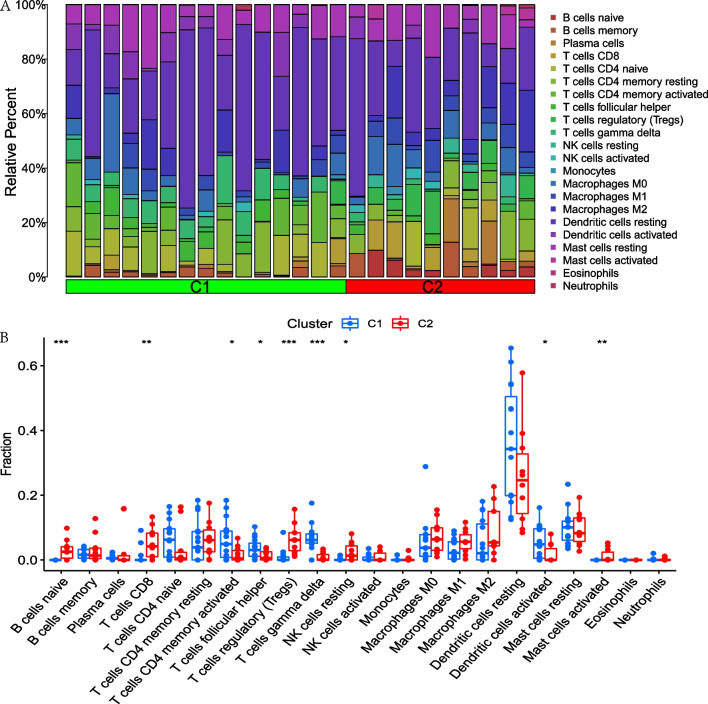
Immune cells infiltration analysis between C1 and C2 module. **A**. Bar graph of immune cells infiltration between C1 and C2 module. **B**. Boxplot showed that 9 immune cells were significantly different between two groups. *p < 0.05; **p < 0.01; ***p < 0.001

The gene modules with different expressing levels of pyroptosis were screened by consistent cluster analysis based on ConsensusClusterPlus package of R language software 4.2.0. The result showed that there was less confounding between the modules, Fig. 4A, with the highest internal consistency greater than 0.8 (Fig. 4C) when the the number of modules was 2. Figure 4B–D. By this method, 15 samples were assigned to C1 module, and 10 samples were assigned to C2 module. Differential expression analysis showed that CHMP2B, GZMB expressed significantly higher in C1 module than C2 module, while IL1B expressed significantly lower in module C1 than module C2. Figure 5A–B. PCA analysis showed that patients were able to well distinguish between C1 and C2 modules based on the expression levels of PRGs. Figure 5C. Immune cells infiltration analysis showed that B cells naive, T cells CD8, T cells CD4 memory activated, T cells follicular helper, T cells regulatory (Tregs), T cells gamma delta, NK cells resting, Dendritic cells activated and Mast cells activated were significantly difference between two modules. Figure 6A–B.

### Weighted gene co-expression analysis of the pyroptosis gene modules

**Fig. 7:**
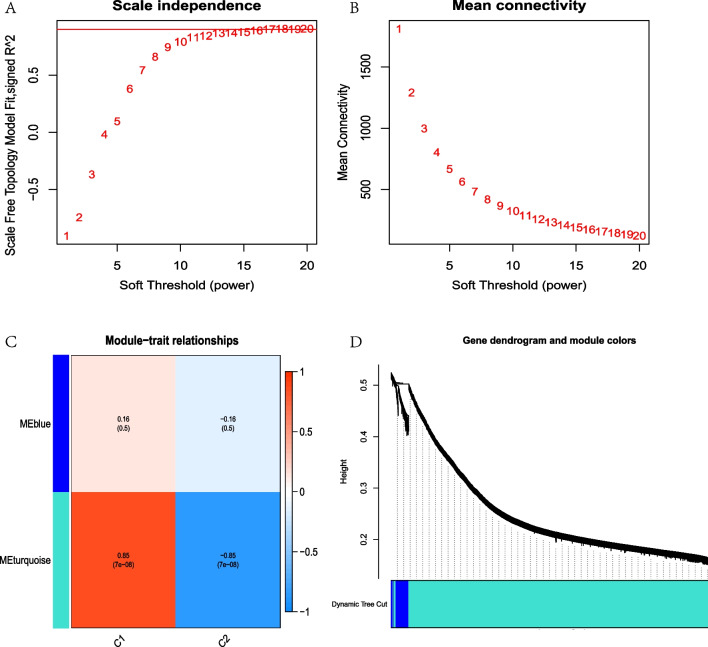
Weighted gene co-expression analysis of the pyroptosis gene modules. **A**–**B**. Screened of soft threshold. **C** WGCNA screened 2 modules, in which turquoise module showed significant difference in C1 and C2 modules with *p* = 7e−08, and correlation coefficient 0.85. **D**. Gene dendrogram and module colors

To further screened out the gene modules with the most significant difference and the highest correlation coefficient between the different pyroptosis gene modules, the WGCNA was performed. The results showed that β = 1 was the best threshold value. Figure 7A–B. In this β = 1, 2 modules with the color of turquoise and blue were identified. To be noted, the turquoise module showed significant difference in C1 and C2 modules with *p* = 7e−08, and correlation coefficient 0.85. Figure 7C–D. To further screened the core genes of the turquoise module, 84 hub PRBMs were selected by setting the criteria of geneSigFilter = 0.85 and moduleSigFilter = 0.95.

### Construction of a disease diagnostic models for AD using machine learning

**Fig. 8:**
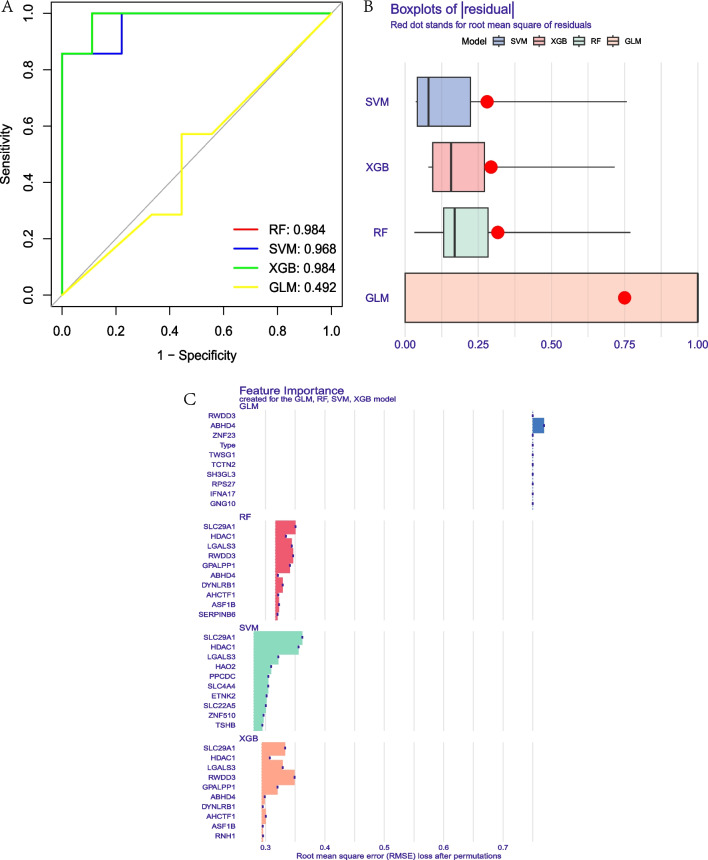
Construction of a disease diagnostic models for AD using machine learning. **A**. The ROC curve showed that the AUC of RF was 0.984, the AUC of SVM was 0.968, the AUC of XGB was 0.984 and the AUC of GLM was 0.492. **B**. Boxplots of residual. **C**. Feature importance of PRBMs of machine models

To construct the AD disease diagnosis model, the RF, SVM, XGB, and GLM machine learning models were constructed. The results showed that the AUC of RF was 0.984, the AUC of SVM was 0.968, the AUC of XGB was 0.984 and the AUC of GLM was 0.492. Figure 8A. The results of residual square showed that the XGB was second to SVM. Figure 8B. Therefore, the XGB model was selected to be the most optimal model and 5 PRBMs HDAC1, GPALPP1, LGALS3, SLC29A1 and RWDD3 with high important scores were persisted to the further analysis. Figure 8C. To be noted, we also performed the RF, SVM, XGB and GLM on the testing datasets GSE32924 and GSE153007. The results showed that the AUC of RF, SVM, XGB and GLM was 1.000 in GSE32924 respectively, and the AUC of RF was 1.000 (Additional file 2: Fig. S1A), the AUC of SVM was 0.857, the AUC of XGB was 0.714 and the AUC of GLM was 0.786 in GSE153007. Additional file 2: Fig. S1B.

### Construction of a nomogram

**Fig. 9:**
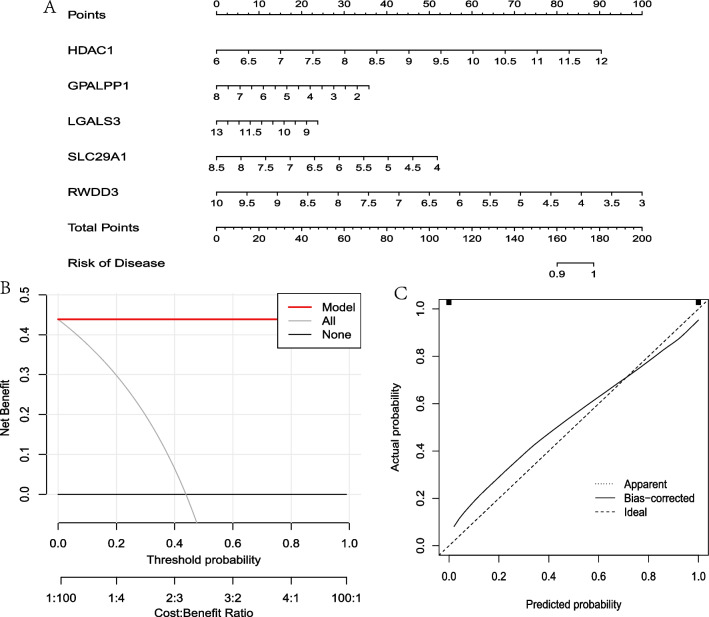
Construction and verification of a nomogram. **A**. Construction of a nomogram based on HDAC1, GPALPP1, LGALS3, SLC29A1 and RWDD3 five PRBMs. **B**. DCA decision curve of the model was completely separated from all curve. **C**. Simulation curve showed that the line of Bias-corrected was closed to the line of Ideal

In order to construct a nomogram that used for the diagnosis of AD, the rms package and rmda package of R language software 4.2.0 were employed. The nomogram was constructed by using HDAC1, GPALPP1, LGALS3, SLC29A1 and RWDD3 five PRBMs. The results of the nomogram showed that there was 100 percent probability to the diagnosis of AD if the score ranked to 180. Figure 9A. The DCA decision curve of the model was completely separated from all curve, which indicated that the AD diagnostic model has a very high reliability. Figure 9B. This result was also verified in the simulation curve. Figure 9C.

### Validation based on external datasets

**Fig. 10:**
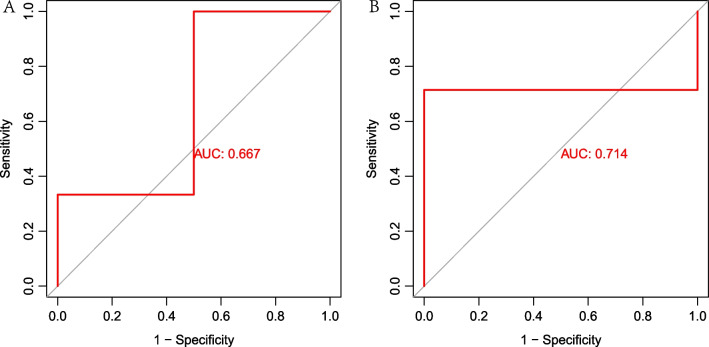
Validation based on external datasets. **A**. The ROC curve showed that AUC of GSE32924 was 0.667. **B**. The ROC curve showed that AUC of GSE153007 was 0.714

The probe matrix of GSE32924 and GSE153007 were transformed to gene matrix by using platform files and Perl language. Next, pROC package was used to verify the efficiency of the model. The results showed the AUC of GSE32924 was 0.667 and the AUC of GSE153007 was 0.714. Figure 10. To be noted, GPALPP1 was lacked in GSE153007. Therefore, we only used the remaining 4 PRBMs as assays. All the above results showed that the efficiency of the model was reliable.

## Discussion

In this study, 22 PRGs were recognized from the training group and 9 DEGs were identified, namely, BAK1, BAX, CASP5, GZMB, IL1A, IL1B, IRF1 (all up-regulated genes) and CHMP2A, CHMP2B (both down-regulated genes). It was worth noting that IL1B, GZMB as well as CHMP2B were also showed significantly different expression in module C1 and C2. Therefore, IL1B, GZMB and CHMP2B were recognized as the three hub PRGs in AD.

IL1 family of cytokines and their receptors were related with various skin disorders, such as AD, allergic contact dermatitis and psoriasis [24]. As a potent pro-inflammation cytokine, IL1B was the best characterized of 11 IL1 family members and crucial to the host-defence responses to pathogens [25]. IL1B and IL1A has been reported increased expression in skin of AD patients with mutations in filaggrin (FLG) and their expression were negatively correlated with the levels of natural moisturizing factors, which was essential for skin barrier [26]. More importantly, the expression of IL1B induced hyper-activation of IL-1R1-expressing mast cells, while the treatment of anti-IL1B antibody can reduce the aggravation of dermatitis in spontaneous mice models of AD with a defective skin barrier [27]. Granzymes B(GZMB) was the most essential member of the Granzymes family, having the strongest apoptotic activity among all granzymes [28]. GZMB was released after the activation of mast cells, leading to cell death and inflammatory processes [29]. The plasma level of GZMB was elevated in AD patients and positively correlated with the severity of pruritus and dermatitis [30]. Moreover, GZMB damaged the skin barrier through E-cadherin and FLG cleavage, which suggested that targeting GZMB may be an effective therapeutic strategy for AD [31]. Charged multi-vesicular body protein 2B (CHMP2B), a component of the heteromer sorting complex III, regulates multi-vesicular biogenesis [32]. The knockdown of CHMP2B can result in abnormal membrane and protein aggregation associated with autophagosomes [33]. Additionally, a failure in the degradation of cardiovascular CHMP2B led to reduced autophagy, aggregation of intracellular proteins, and apoptosis of cardiomyocytes [34]. Notably, study suggested that autophagy linked to negatively regulate pyroptosis and autophagosomes may target inflammasomes for degradation [35]. The ROS-activated autophagy mechanism may represent a negative feedback mechanism to limit the activation of ROS-regulated caspase-1 while clearing ROS damaged organelles and proteins and inhibiting intracellular pathogens [36]. Consequently, it was interesting to investigate the function of CHMP2B in AD from the perspective of mutual regulation of autophagy and pyroptosis.

The correlation between immune cells infiltration and 9 PRGs were analyzed and results revealed that except for CHMP2A, the expressions of the remaining 8 PRGs were correlated with immune cells infiltration in AD. Of all immune cells, T cells CD4 memory activated and Dendritic cells activated aroused our attention for these immune cells showed a significantly different level between normal human and AD patients and C1 and C2 module. Therefore, we focused on the mechanisms of hub PRGs related with Dendritic cells (DCs) and T cells CD4 memory activated.

AD was a Th2/Th22 dominant disease regulated by DC-induced T cell polarization and recruitment of chemokines to specific T cell subsets [37]. Activated DCs migrates to regional lymph nodes where they promoted Th2-related cytokines production and subsequent B-cell IgE conversion [38]. Interestingly, CD4+ memory Th2 cells are most abundant in inflamed lessions [14] and blocking the antigen presents to CD4+ memory T-cells by a humanized monoclonal antibody has demonstrated to be effective in the treatment of AD [39]. In fact, allergens and Staphylococcus aureus entered into skin through impaired epidermis barrier and activated pathogenic skin-homing CLA + CD4+ CCR4+ memory T cells, leading to production of type 2 cytokines, such as IL-4, IL-13, IL-31, and IL-22 [40]. In the present study, GZMB was positively correlated with the DCs activated and T cells CD4 memory activated and IL1B was negatively related to DCs activated. Previous study elucidated that GZMB played a vital role as inducers of Ag cross-presentation by DCs which contributed to immunogenic cell death [41]. CD4 T cell help selectively accelarated the rapid upregulation of GZMB in liver sinusoidal endothelial cells [42]. Moreover, TLSP activated DCs in AD, inducing the differentiation of CD4+ T cells into TH2 cells, which enhanced the inflammation process. Meanwhile, DC-derived TSLP negatively modulated IL-1B and HIF-1α during dectin-1 signaling and this mechanism regulated via dampening Syk phosphorylation [43].

Surprisingly, our results of the nomogram demonstrated that there was 100 percent probability to the diagnosis of AD if the 5 genes HDAC1, LGALS3, SLC29A1, RWDD3 and GPALPP1 totally scores ranked to 180. Among them, only HDAC1 expression was positively correlated with the risk of developing AD, and the remaining four were negatively correlated. Hence, understanding the functions of 5 genes may provide prospective insight in the regulation of AD immune-inflammatory signals.

Previous studies have found that the activation of histone deacetylase(HDAC) was essential for epidermal differentiation [44, 45] and HDAC1 was involved in FLG reduction in AD [46]. FLG, a crucial structural protein for skin barrier, is down-regulated in chronic inflammatory environments [47]. TNFα + IFNγ stimulation repressed FLG expression by promoting the FRA1:c-JUN:HDAC1 complex and knockdown of HDAC1 abolished the inhibition of TNFα + IFNγ on FLG expression in keratinocyte [46]. Moreover, the expressions of HDAC1, FRA1 and c-JUN were increased in 2,4-dinitrochlorobenzene induced AD-like mouse models [46]. Taken together, downregulating the expression of HDAC1 may contribute to the amelioration of skin disorders in AD. Galectin-3(Gal-3), also known as LGALS3, plays a critical role in self-tolerance, inflammation, and fibrosis [48]. It has reported that the level of Gal-3 was decreased in AD epidermis compared with control samples. In vivo, the release of IL-6 in IL-4-stimulated keratinocytes was downregulated by Gal-3 [49]. Solute Carrier Family 29 Member 1 (SLC29A1) was point as a top novel gene for early allergic sensitization [50]. In addition, facilitated influx of adenosine through equilibrative nucleoside transporter 1 (ENT1/SLC29A1) was necessary for the inhibition of FcεRI-mediated degranulation in mast cells [51]. Contemporary, the degranulation of mast cells can release the allergenic mediators, such as histamine, leukotriene, and inflammatory cytokines, which cause itching in AD [52, 53]. RSUME (for RWD-domain-containing sumoylation enhancer), also known as RWDD3, was identified as a key mediators of inflammatory regulation [54]. RSUME enhanced sumoylation of Iκβ, leading to the restraint of NF-κβ activity on two renowned inflammatory genes, cyclooxygenase-2 and IL-8 [55]. GPALPP Motifs Containing 1 (GPALPP1) was first reported to be a new biomarker for the diagnosis of AD in current study. Collectively, RWDD3, SLC29A1, LGALS3 and GPALPP1 genes were associated with allergic or inflammatory diseases, which may show beneficial effect for AD.

## Conclusion

In conclusion, in this study, 5 novel PRBMs of AD were identified and used to establish a nomogram for the accurate diagnosis of AD. Our results may provide a foundation for the further study of pyroptosis on the occurence and development of AD.

## Supplementary Information

**Additional file 1**: **Table S1**. Pyroptosis related genes.

**Additional file 2**: **Fig. S1**. Validation of machine learning based on external datasets. A. The ROC curve showed that the AUC of RF, SVM, XGB and GLM was 1.000 in GSE32924. B. The ROC curve showed that the AUC of  RF was 1.000, the AUC of SVM was 0.857, the AUC of XGB was 0.714 and the AUC of GLM was 0.786 in GSE153007.

## Data Availability

The data of this study were acquired from GEO database (https://www.ncbi.nlm.nih.gov/geo/) and MSigDB database (https://www.gsea-msigdb.org/gsea/index.jsp).

## Abbreviations

AD: Atopic dermatitis
PRBMs: Pyroptosis related biological markers
PRGs: Pyroptosis related genes
MSigDB: Molecular signatures database
GEO: Gene expression omnibus
DEGs: Differentially expressed genes
WGCNA: Weighted correlation network analysis
RF: Random forest
SVM: Support vector machines
XGB: Extreme gradient boosting
GLM: Generalized linear model
ROC: Receiver operating characteristic
AUC: Area under curve
DCA: Decision curve analysis
DCs: Dendritic cells
NKs: Natural killer cells
GSDMD: Gasdermin D
ML: Machine learning
PCA: Principal component analysis
ASC: Apoptosis-associated speck-like protein containing a caspase-recruiting domain
NLRP3: NLR family pyrin domain containing 3
AIM2: Absent in melanoma-2
ML: Machine learning
CA: Principal component analysis
Tregs: T cells regulatory
FLG: Filaggrin
GZMB: Granzymes B
CHMP2B: Charged multi-vesicular body protein 2B
HDAC: Histone deacetylase
Gal-3: Galectin-3
SLC29A1: Solute carrier family 29 member 1
GPALPP1: GPALPP motifs containing 1
