# Supplementary material for: Construction and verification of atopic dermatitis diagnostic model based on pyroptosis related biological markers using machine learning methods
Source: BMC Med Genomics. 2023 Jun 17;16:138. doi: 10.1186/s12920-023-01552-5 (PMC10276470; doi:10.1186/s12920-023-01552-5)
Supplement: Supplementary file 2 — Additional file 2: Fig. S1. Validation of machine learning based on external datasets. A. The ROC curve showed that the AUC of RF, SVM, XGB and GLM was 1.000 in GSE32924. B. The ROC curve showed that the AUC of RF was 1.000, the AUC of SVM was 0.857, the AUC of XGB was 0.714 and the AUC of GLM was 0.786 in GSE153007. [file 12920_2023_1552_MOESM2_ESM.pdf]

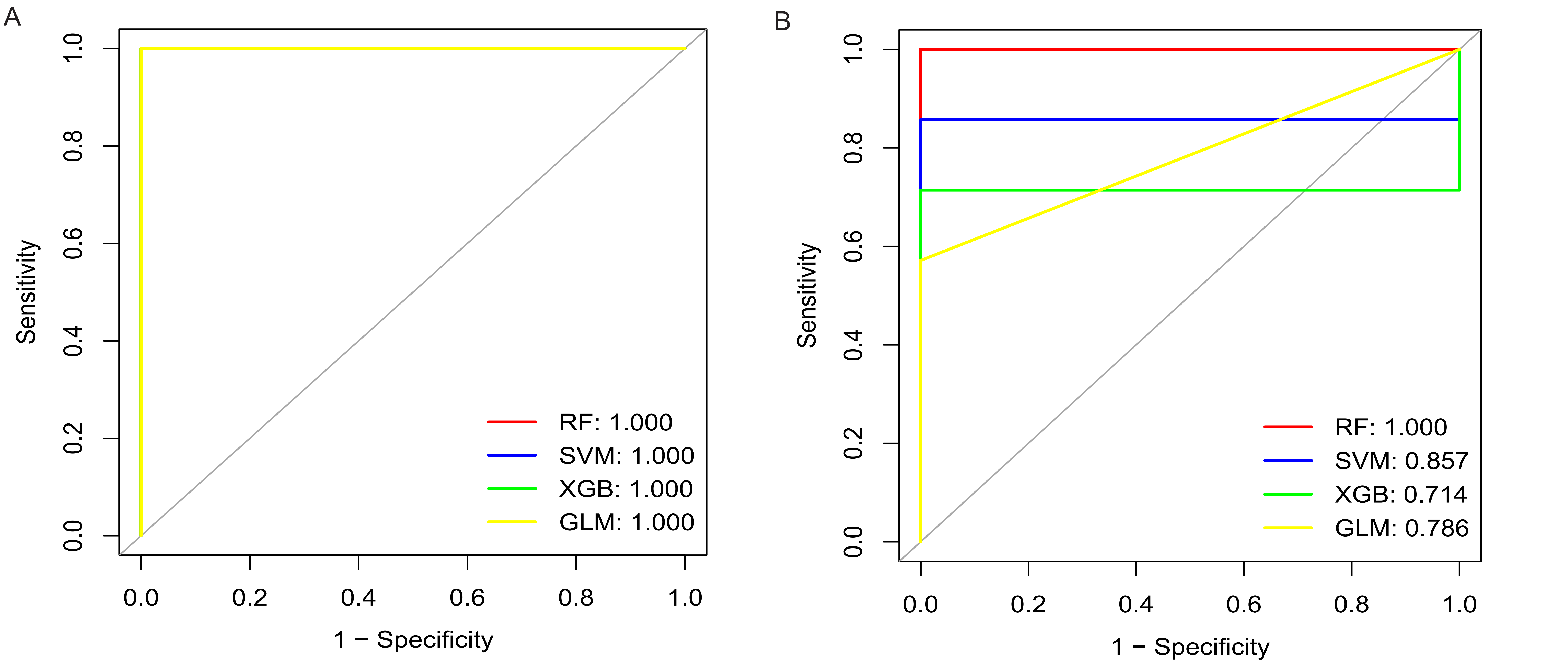

Supplemental Figure1. Validation of machine learning based on external datasets. A. The ROC curve showed that the AUC of RF, SVM, XGB and GLM was 1.000 in GSE32924. B.The ROC curve showed that the AUC of RF was 1.000, the AUC of SVM was 0.857, the AUC of XGB was 0.714 and the AUC of GLM was 0.786 in GSE153007.
